# 

**Published:** 1879-09

**Authors:** 


					To Readers and Correspondents.
Communications intended for publication, and exchange journals and papers
should be addressed to the Editor of the American Journal of Denta:
Science, 259 N. Eutaw St., Baltimore, Md. All letters relating to the business
of the journal, or containing cash remittances, to be directed to Messrs. Snowden
& Oow^ian. Articles for publication should be received by the editor at leas]
three weeks previous to the first d^y of the month on which the number in whicl
it is designed for them to appear, is issued.
SCT'The American Journal of Dental Science will contain fiftj pa^es of
reading matter in each number, making a volume of about
Six Hundred pages
0F advertisements.
Ml i
T H E
AMERICAN JOURNAL
OF
DENTAL SCIENCE.
The Journal is issued on the first of each month and contains not less
than fifty pages of reading matter in each number, exclusive of adver-
tisements, making a volume of six hundred pages per annum.
In each number will be found Original Communications, Corres-
pondence, Selected Articles, Monthly Summary, Bibliographical No-
tices and Editorial Matter, and every effort will bp exerted to render
the Journal worthy of the patronage of the Dental profession.
A generous co-operation is respectfully solicited from the members
of the profession; and as the Journal has now a printing office of its
own, which materially lessens the cost of its publication, it will be
issued at the reduced subscription price of
$2.50 For Annum in Advance.
Communications are respectfully solicited on all subjects pertain -4
ing to the practice of dentistry, as well as reports of cases occurring
in dental practice. WM
??'*1
RATES OF ADVERTISING. #g
1 MO. 2 MO. 3 MO. 6 MO.
;15 00 $22 50 $30 00 $50 00
10 00 15 00 20 00 30 00 J<50 0'(
7 00 11 00 15 00 20 00 '30 0(
5 00 8 00 11 00 15 00 |jg^20 00
All original communications designed for publication',
review, new instruments for notice, exchanges, &c., should b
to the editor, 259 N. Eutaw St., Baltimore, Md.
All advertisements and subscriptions should be
SNOWDEN & CO^IA
Publishers of the Am. Journal of Dental T _
86 W. Fayette St. Bet. Charles M Liberty S

				

